# Preparation of Itaconic Acid-Modified Guar Gum Composite Hydrogel for Water Retention in Mine Ecological Restoration

**DOI:** 10.3390/gels12100921

**Published:** 2026-10-14

**Authors:** Shixue He, Ting Zhao, Daning Lang, Ronglan Wu

**Affiliations:** 1 Key Laboratory of Oil & Gas Fine Chemicals, Ministry of Education & Xinjiang Uygur Autonomous Region, School of Chemical Engineering, Xinjiang University, Urumqi 830017, China; 2 School of Mechanical Engineering & Center for Post-Doctoral Studies of Mechanical Engineering, Xinjiang University, Urumqi 830017, China; 3 Department of Energy and Chemical Engineering, Xinxing Vocational and Technical College, Xinxing 839000, China

**Keywords:** guar gum, itaconic acid, composite hydrogel, mine soil, water retention

## Abstract

To tackle poor water retention in degraded mine soil and the low stability and short water-holding duration of natural polymer hydrogels, an itaconic acid-modified guar gum composite hydrogel (IAGG hydrogel) was developed using guar gum (GG) as the polymer substrate and itaconic acid (IA) as the functional monomer. This study aimed to improve the stability, water-retention duration, and water-absorption performance of GG-based hydrogels through a covalent–dynamic multiple-crosslinking design and to evaluate their potential for mine ecological restoration. The hydrogel network was constructed through IA-related covalent interactions, ECH-mediated ether crosslinking, and borax-mediated dynamic interactions, forming a covalent–dynamic multiple-crosslinked structure. IA-introduced carboxyl groups enhanced hydrophilicity and water retention via hydrogen bonding and ionic hydration, while covalent–dynamic crosslinking synergy improved network stability and wet–dry cycle resistance. Under the optimized preparation conditions, the IAGG hydrogel exhibited a water absorption capacity of 370.7 g/g in deionized water, and the I5G5 hydrogel retained a water absorption capacity of 255.4 g/g after 10 swelling–dehydration cycles. Soil experiments showed that IAGG increased mine soil water-holding capacity from 13.3 to 77.6 g/g, extended retention from 5 to 14 days, and supported the germination and subsequent seedling development of *Suaeda salsa*. After 4 months of soil burial, the hydrogel exhibited approximately 63% weight loss, indicating substantial structural deterioration under the tested soil-burial conditions. These results demonstrate the potential of IAGG as a water-retention material for mine ecological restoration.

## 1. Introduction

Soil degradation caused by mining has become one of the core challenges in the field of global ecological restoration, and the lack of soil water retention capacity is the key bottleneck restricting mine ecological restoration [1]. Both open-pit mining and underground mining severely disturb the topsoil, resulting in vegetation destruction, soil structure fragmentation, porosity decline, and organic matter loss, leading to the disintegration of soil aggregate structure and the reduction of water-holding pores, thus leading to a sharp decline in soil water-holding performance [2]. Degraded soil not only makes it difficult to maintain plant root growth and water absorption, but also easily induces ecological problems such as soil erosion, desertification, and secondary salinization under rainfall erosion, forming a vicious cycle of “water shortage-poor vegetation establishment-further degradation” [3]. According to statistics, the area of degraded land caused by mining in China has reached millions of hectares, and it is increasing at the rate of about tens of thousands of hectares every year. These areas are generally faced with practical difficulties such as water shortage, difficulty in vegetation restoration, and weak self-repair ability of ecosystems [4,5]. Therefore, it is of great practical significance and application value to develop efficient and environmentally friendly soil water-retaining materials and improve the water-retaining capacity of degraded soil in mines, which will promote mine ecological restoration and regional sustainable development.

Among many water-retaining materials, polymer hydrogels are regarded as one of the most promising soil water-retaining agents because of their three-dimensional crosslinking networks, which can absorb and retain water amounts hundreds of times their own weight and slowly release it under drought conditions [6]. Among them, GG, as a widely available and biodegradable natural polysaccharide [7,8], is rich in hydroxyl groups in its molecular chain, which endows it with good soil water retention capacity [9]. For example, Cui et al. used quaternary ammonium guar gum and humic acid as raw materials to prepare a superabsorbent hydrogel through physical and chemical crosslinking, and the hydrogel contained four hydrophilic groups (quaternary ammonium salt, hydroxyl group, carboxyl group, and ketone group), which can more than double the water retention capacity of soil [10]. Moreover, GG and borax can form dynamic borate bonds, which allow the prepared hydrogel to have the characteristics of a simple process and environmental friendliness [11]. However, the mechanical strength of the network structure crosslinked by a single dynamic borate bond is low, and it is easily destroyed by microbial degradation, dry–wet cycle stress, and root mechanical action in a complex soil environment, resulting in short water-holding time and rapid attenuation of the water-holding effect, which makes it difficult to support the water demand of vegetation in the whole growth cycle. Meanwhile, pure GG hydrogel has a single kind of hydrophilic group, limited water absorption capacity and swelling durability, and its water absorption performance is easily inhibited by the ion shielding effect in saline and alkaline mine soil environments, so it is difficult to meet the long-term water retention requirements in mine drought environments [12,13,14]. Therefore, it is urgent to introduce green and efficient functional monomers for chemical modification and structural optimization of GG hydrogel, improve its structural stability and environmental adaptability by constructing a covalent–dynamic multiple crosslinking network, and introduce strong hydrophilic groups to enhance its water absorption and storage capacity, thus breaking through the application limitations of existing materials in mine ecological restoration.

IA is an unsaturated dicarboxylic acid derived from biomass fermentation. Its molecular structure contains an active carbon–carbon double bond (C=C) and two carboxyl groups (-COOH) [15], and it is listed as one of the most promising biomass-based platform compounds by the US Department of Energy. The unsaturated structure of IA provides reactive functionality for chemical modification, while its carboxyl groups can participate in reactions or interactions with hydroxyl-containing polymer chains, thereby contributing to network construction. As strong hydrophilic groups, carboxyl groups can ionize to produce -COO^−^ in aqueous solution and combine with a large number of water molecules through hydrogen bonding and ionic hydration, which can enhance the water absorption and water-retention capacity of hydrogels [16]. Meanwhile, -COO^−^ can form electrostatic interactions or coordination complexes with metal cations such as Ca^2+^, Fe^3+,^ and Al^3+^ on the surface of soil particles, thus enhancing the adhesion and antiscouring ability of hydrogels in soil and reducing the loss of water and fertilizer [17]. In addition, IA has the advantages of good biocompatibility and low cost [18]. These characteristics make IA a promising functional component for environmentally oriented material systems. Its combination with the natural polymer GG is therefore suitable for the development of hydrogel materials for ecological restoration applications.

Based on this, an itaconic acid-modified guar gum composite hydrogel (IAGG hydrogel) was prepared using GG as the polymer substrate, IA as the functional monomer, ECH as the chemical crosslinking agent, and sodium tetraborate decahydrate as the dynamic crosslinking component. The effects of technological parameters such as raw material ratio, crosslinking agent dosage, and reaction temperature on the water absorption performance of the hydrogel were systematically investigated to determine the optimal preparation conditions. The microstructure, chemical structure, thermal stability, and viscoelasticity were characterized by scanning electron microscopy (SEM), Fourier transform infrared spectroscopy (FTIR), thermogravimetric and derivative thermogravimetric analysis (TG/DTG), and rheological measurements. We further evaluated its water absorption behavior and recycling stability in different aqueous environments (pH and salt concentration), together with water retention, seed germination and seedling development, and soil-burial degradation behavior in mine soil. The purpose of this study was to improve the stability, water-retention duration, and water-absorption performance of GG-based hydrogels through a covalent–dynamic multiple-crosslinking design and to evaluate their potential for mine ecological restoration.

## 2. Results and Discussion

### 2.1. Morphology and Physicochemical Characterization

**Figure 1:**
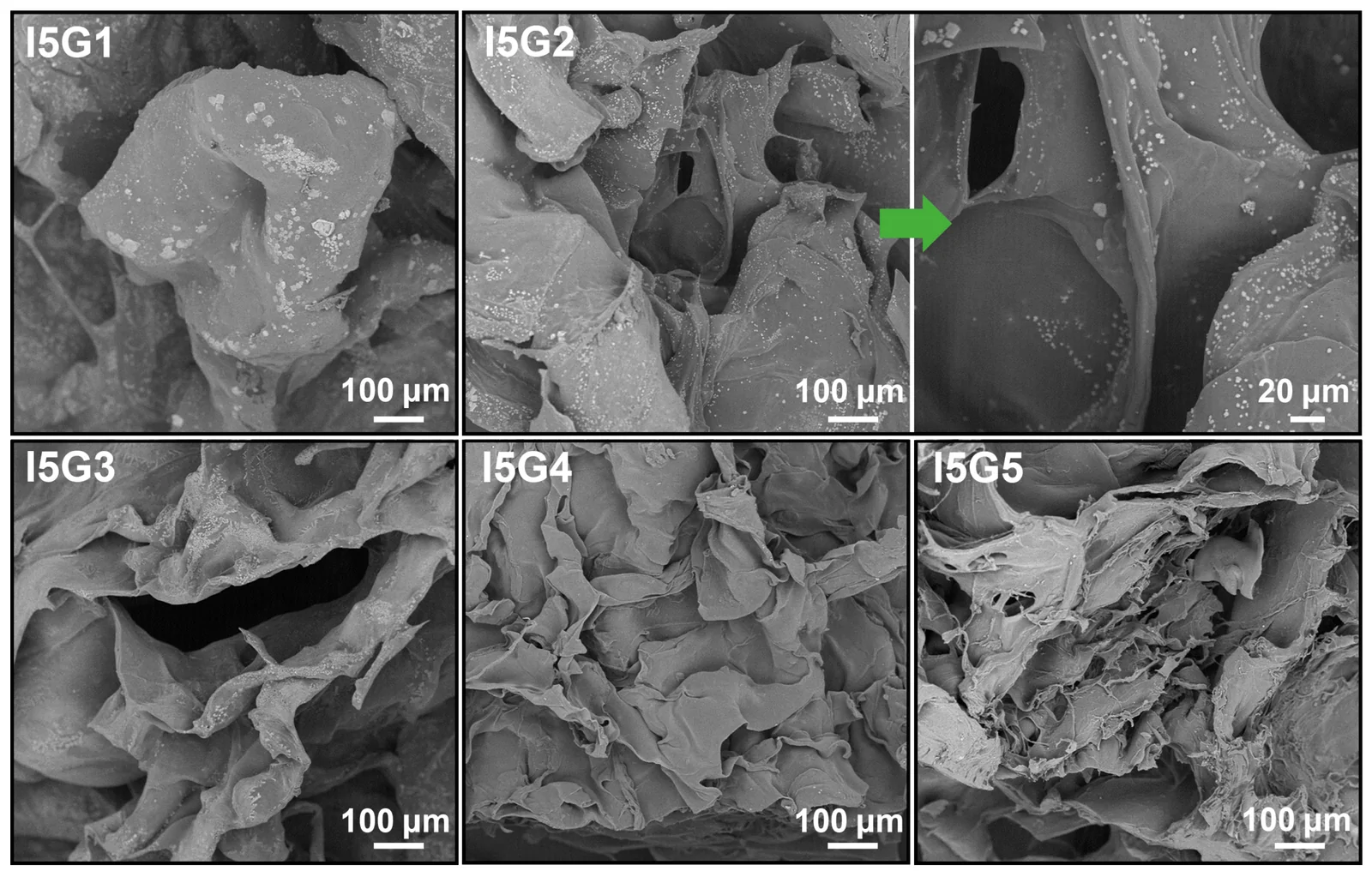
SEM images of IAGG hydrogels with different IA/GG mass ratios.

Figure 1 shows that as the GG proportion increases from I5G1 to I5G5, the morphology of the IAGG hydrogel exhibits a gradual evolution from dense blocks to a loose, porous three-dimensional network, reflecting the influence of the raw-material ratio on the crosslinked network structure. When the mass ratio of IA to GG is 5:1, the surface of I5G1 is smooth and compact, and only a few micropores and incompletely reacted granular residues are found. The observed dense morphology at low GG content may be related to the limited number of hydroxyl-containing interaction sites and tighter molecular-chain packing [19]. When the GG ratio rises to 5:2, initial wrinkles and concavities appear on the surface of I5G2, and the number of pores increases, while a lamellar structure begins to form locally. At high magnification, particles remain attached to the lamellar surface, suggesting a transition from a relatively dense structure toward a looser network.

When the GG ratio is further increased to 5:3 and 5:4, the surface wrinkles and lamellar features become progressively more pronounced, and the number and size of pores gradually increase. Among them, I5G3 is mainly composed of large cavities and laminated sheets, and the edges of the sheets are obviously curled; I5G4 folds are denser and more finely divided, and the hole walls become thinner and overlap with each other, and the whole structure changes from loose lamellae to a continuous network. This evolution may be related to the increased availability of GG hydroxyl groups, which may promote ECH-mediated ether crosslinking and borax-mediated dynamic interactions, thereby increasing network branching and interlamellar spacing [20].

When the ratio of IA to GG is 5:5, I5G5 presents a highly pleated interpenetrating lamellar structure, forming a continuous and open three-dimensional porous network. The abundant interaction sites provided by the higher GG content, together with the crosslinking agents, may strengthen intermolecular connections and mutual support between lamellae, resulting in a more open porous microstructure [21]. The observed morphological evolution indicates that the raw-material ratio influences the network architecture: a more open porous structure can facilitate water diffusion and storage, while a continuous interpenetrating skeleton may contribute to mechanical stability [22].

**Figure 2:**
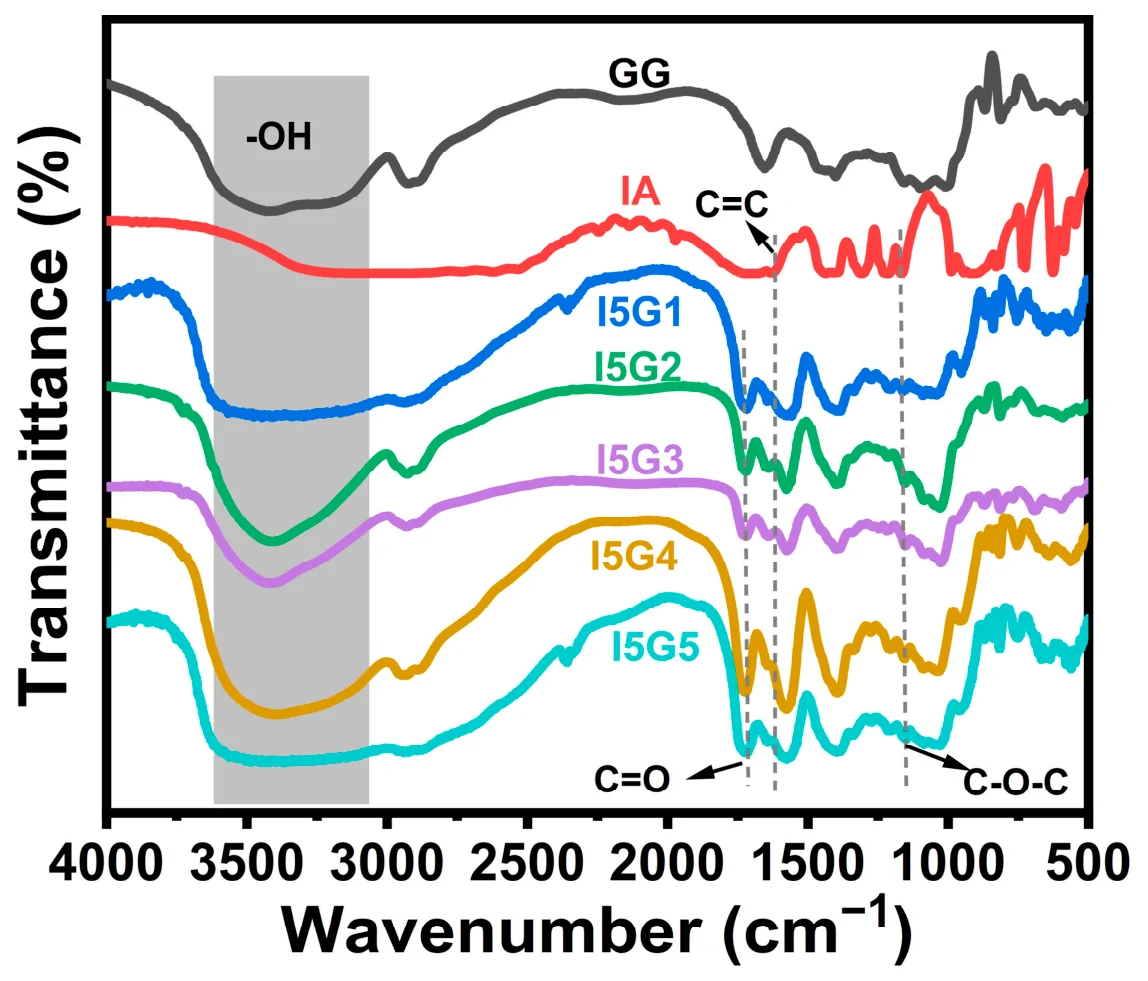
FTIR spectra of GG, IA, and IAGG hydrogels with different IA/GG mass ratios.

It can be seen from Figure 2 that the broad absorption peak of GG at 3600–3000 cm^−1^ is the polysaccharide-OH stretching vibration [23], 2928 cm^−1^ is the sugar ring C-H stretching vibration, 1642 cm^−1^ is the adsorbed water H-O-H bending vibration, 1416 cm^−1^ corresponds to C-H bending vibration, and 1156 cm^−1^, 1080 cm^−1^, and 1028 cm^−1^, respectively, correspond to C-O-C stretching vibrations. The broad peaks of IA correspond to O-H stretching vibrations in -COOH in the 3600–2500 cm^−1^ range (due to the broadening of intermolecular hydrogen bonds), the C=O stretching vibration at 1712 cm^−1^, the C=C stretching vibration at 1632 cm^−1^, in-plane bending and C-O stretching vibrations at 1412 cm^−1^ and 1302 cm^−1^, respectively, and the double bond C-H bending vibration at 932 cm^−1^.

The FTIR spectra of I5G1–I5G5 show similar overall profiles, and with the increase in the GG ratio, the 1725 cm^−1^ ester carbonyl peak and 1150 cm^−1^ ether bond peak gradually increase, suggesting changes in ester- and ether-related structures as the GG content increases. The hydroxyl peak at 3600–3000 cm^−1^ is obviously narrower than that of GG, indicating that some hydroxyl groups participated in the etherification of ECH and the esterification of IA carboxyl groups, and the original intermolecular hydrogen bond network was remodeled. The carbonyl-related band near 1725 cm^−1^ [24] is consistent with the formation of ester-related structures involving GG hydroxyl groups and IA-derived carboxyl groups. The C=C characteristic peak of IA at 1632 cm^−1^ basically disappears (the remaining weak absorption is due to adsorbed water), indicating substantial consumption or transformation of the unsaturated groups during hydrogel formation. At 1150 cm^−1^, the C-O-C ether bond peak is significantly enhanced, which is consistent with ECH-mediated ether crosslinking [25]. The 1020 cm^−1^ sugar ring skeleton peak remains, which indicates that the main polysaccharide backbone of GG is largely retained in the reaction. Overall, the FTIR results support the formation of a multiple-crosslinked IAGG network containing ester- and ether-related structures, while borax is considered to contribute dynamic interactions within the network.

**Figure 3:**
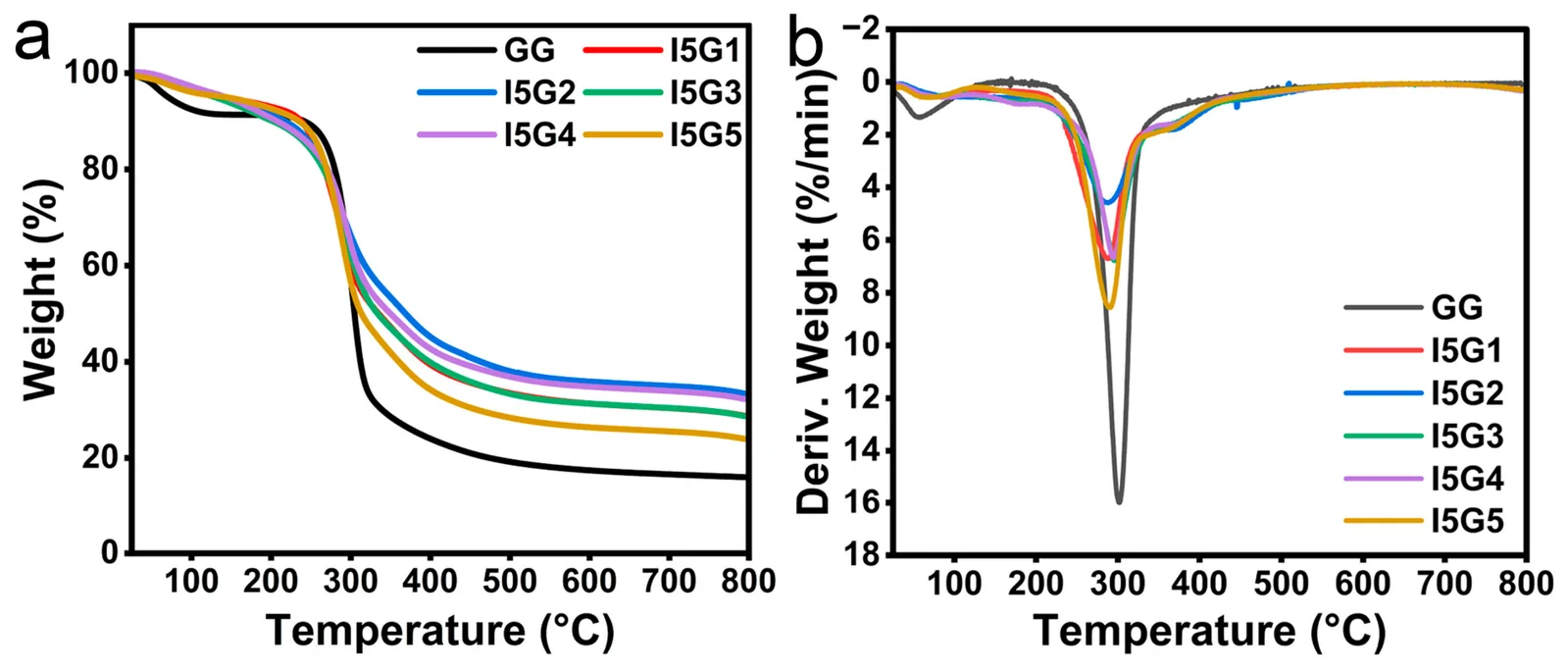
(**a**) TG and (**b**) DTG curves of IAGG hydrogel.

According to Figure 3a, the thermal decomposition process of the IAGG hydrogel can be divided into three stages. In the first stage (25–150 °C), all samples show a mass loss of 3–5%, which is due to the evaporation of adsorbed water and bound water. The second stage (200–400 °C) is the main decomposition stage, which corresponds to polymer backbone fracture, glycosidic bond cleavage, and crosslinked network degradation. The decomposition of pure GG is the fastest at this stage, and the mass retention rate is only 42% at 300 °C, while that of I5G5 can reach 68%, which indicates that the introduction of a crosslinked network significantly delays the thermal decomposition of the main chain. In the third stage (400–800 °C), the mass-loss curves gradually level off, mainly due to the further oxidation and decomposition of the residual carbon skeleton [26]. The final residual rate of I5G5 is the highest, indicating that its crosslinked network has better thermal stability at high temperatures [27].

Moreover, the main decomposition peaks of all samples are concentrated in the range of 300–350 °C (Figure 3b). Pure GG has the sharpest decomposition peak and the lowest peak temperature (312 °C), the highest decomposition rate, and the lowest thermal stability. With the increase in the GG ratio, the peak temperature of the decomposition peak of the hydrogel gradually increases, and the peaks broaden. This phenomenon is attributed to the synergistic effect of multiple crosslinking networks in the system: the esterification reaction between IA and GG hydroxyl groups introduces ester bond crosslinking points, the etherification crosslinking of ECH enhances the covalent connection between molecular chains, and borax and GG hydroxyl groups form dynamic borate crosslinking points; with the increase in the GG ratio, the number of hydroxyl groups that can participate in crosslinking increases, the density of covalent crosslinking and dynamic crosslinking increases synchronously, the movement of molecular chains is limited, and the main chain needs to be broken at a higher temperature, thus delaying the thermal decomposition rate.

**Figure 4:**
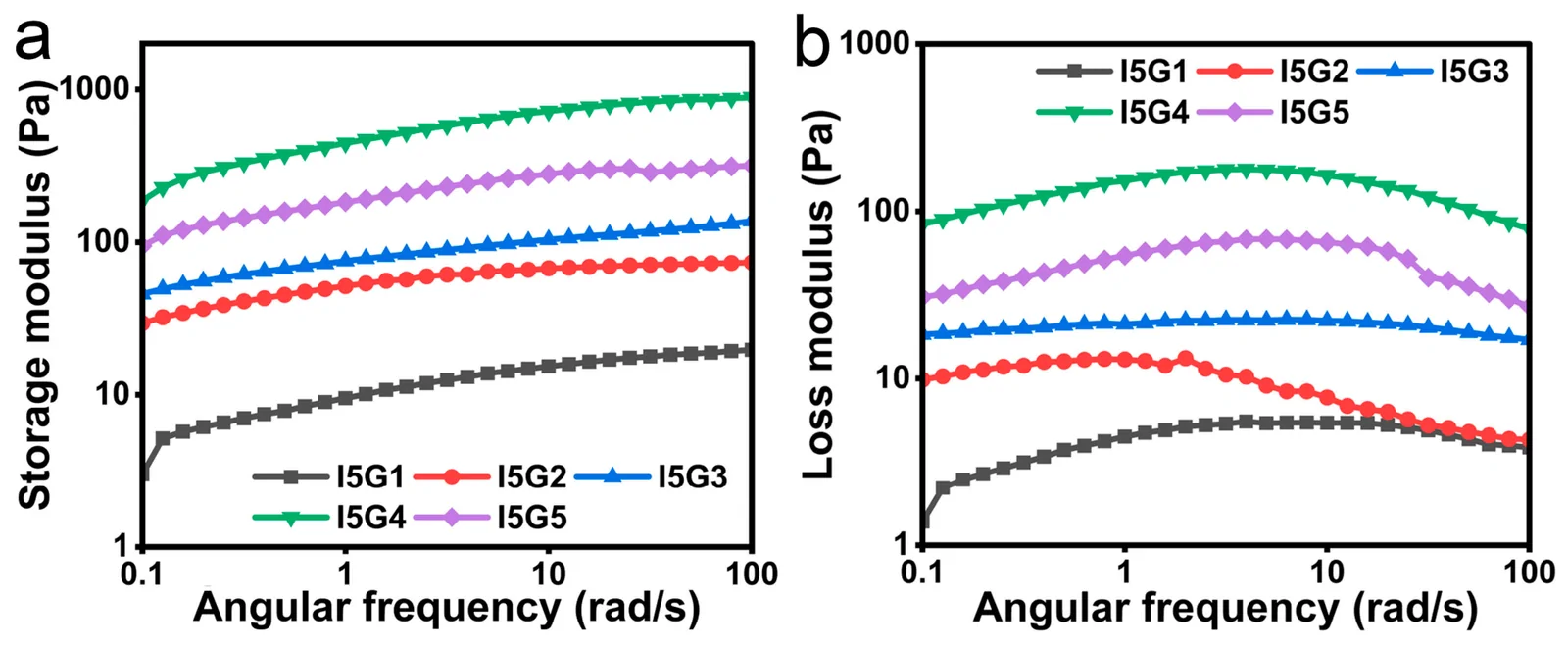
(**a**) Storage modulus and (**b**) Loss modulus of IAGG hydrogel.

In addition, the storage modulus (G′) of all hydrogels increases with the increase of angular frequency (ω), and G′ is greater than the loss modulus G″ (Figure 4a), showing an elastic-dominated gel behavior, indicating that they have a stable three-dimensional network structure. With the increase of the GG ratio from 5:1 to 5:4, the G′ value of the hydrogel increases significantly, indicating that increasing the GG ratio can increase the density of the crosslinked network and enhance the elasticity. At the same time, the growth trend of G′ gradually slows down with the increase of ω, which reflects that the network structure has certain anti-deformation ability. The G′ of I5G5 is slightly lower than that of I5G4, which may be caused by excessive GG leading to chain entanglement saturation or lower crosslinking efficiency. With the increase of ω, the loss modulus G″ first increases and then decreases, with the peak value of G″ in I5G4 being the highest (212 Pa) and that in I5G1 being the lowest (Figure 4b), indicating that a higher GG ratio will increase the viscous dissipation in the network [28]. The G′ of all hydrogels is always greater than G″, and the gap between them increases with the increase of GG ratio, suggesting that hydrogels with higher GG proportions exhibit greater elasticity and a more stable network structure. This rheological behavior stems from the synergistic effect of multiple crosslinked networks in the system [29]: with the increase of the GG ratio, the number of hydroxyl groups that can participate in crosslinking increases, and the density of covalent crosslinking and dynamic crosslinking increases simultaneously, which enhances the elasticity and viscous dissipation of the network. I5G4 shows the best balance between elasticity and viscosity, which indicates that the viscoelasticity of the hydrogel can be effectively optimized by adjusting the ratio of GG to IA.

### 2.2. Water Absorption Performance of IAGG Hydrogel

**Figure 5:**
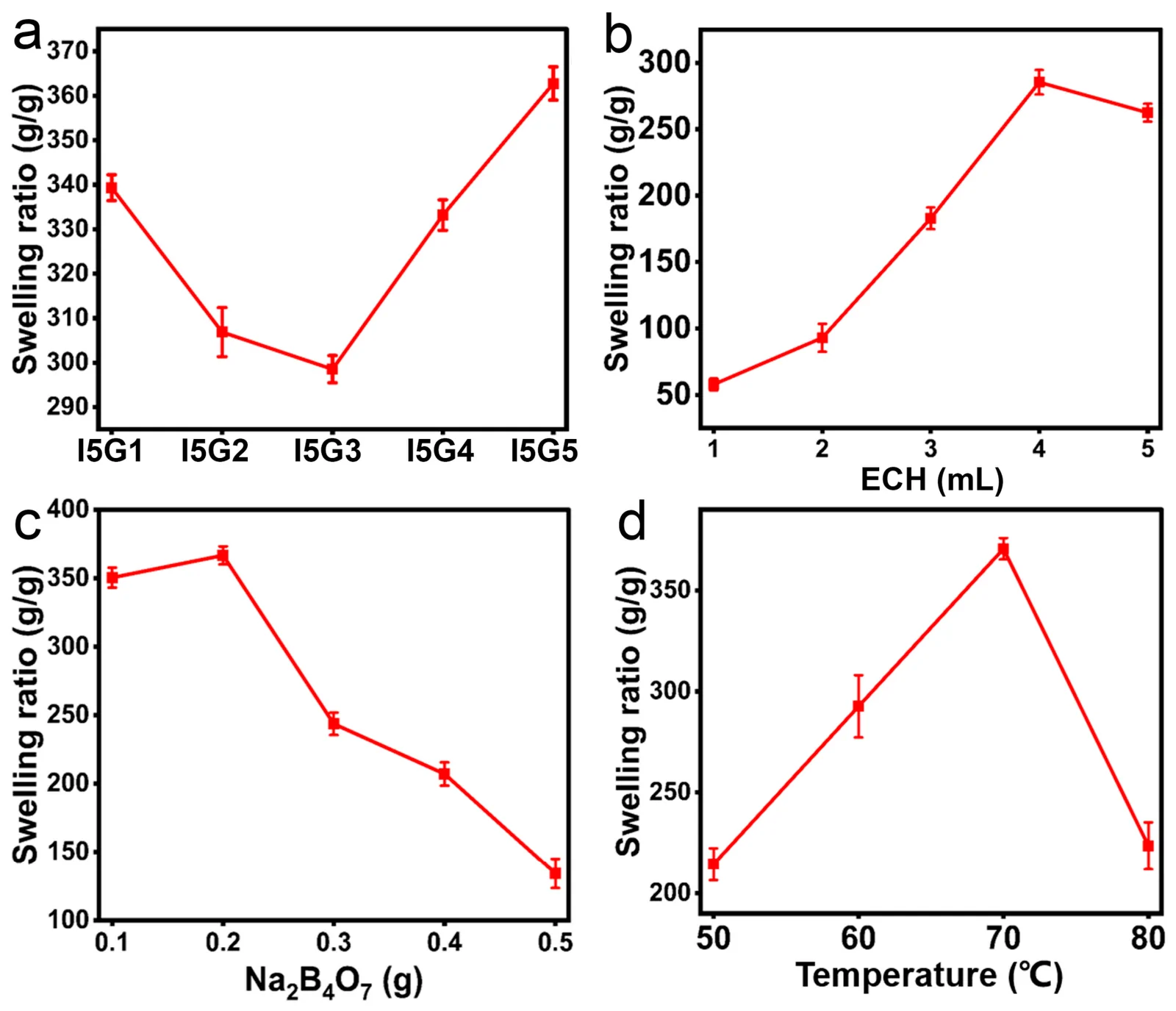
Water absorption of IAGG hydrogels under different preparation conditions: (**a**) raw material ratio; (**b**) amount of ECH; (**c**) amount of Na_2_B_4_O_7_·10H_2_O; (**d**) temperature.

As can be seen from Figure 5a, with the increase in GG ratio, the water absorption of IAGG hydrogels first decreases and then increases: I5G1 is 340.2 g/g, I5G3 decreases to a minimum of 298.4 g/g, and I5G5 increases to a maximum of 370.7 g/g. In the low GG range (5:1–5:3), the synergistic crosslinking of IA, ECH, and borax makes the network compact; the porosity decreases, the penetration of water molecules is limited, and the water absorption decreases. Meanwhile, the concentration difference between the hydrogel interior and the external solution generates osmotic pressure that promotes water absorption [30]. In the high GG range (5:3–5:5), porosity and hydrophilicity become the dominant factors, and a large number of hydroxyl groups introduced by excessive GG enhance hydrophilicity and promote the formation of more folds and holes in the network, thus increasing the water-holding space [31]; finally, the water absorption capacity exceeds the initial level. As a chemical crosslinking agent, the amount of ECH may regulate the network structure and water absorption performance of the hydrogel (Figure 5b). When the dosage of ECH is increased from 1 mL to 5 mL, the water absorption capacity first increases and then decreases. This trend may be related to changes in the number of crosslinking points and network structure. At relatively low ECH dosages, the number of crosslinking points may increase, and the network structure may become more favorable for maintaining structural integrity while retaining sufficient hydrophilic groups to bind water molecules through hydrogen bonding and electrostatic interaction.

However, when the dosage of ECH is increased from 4 mL to 5 mL, an excessive amount of crosslinking agent may lead to an excessively high crosslinking degree and tighter network pores, while some hydrophilic groups may be consumed, which may weaken the hydration ability and limit the entry of water molecules, ultimately reducing water absorption; the overall trend first increases and then decreases. This phenomenon may be attributed to the balance between the hydration of hydrophilic groups and the spatial limitation of crosslinked networks. In the IAGG hydrogel system, Na_2_B_4_O_7_·10H_2_O is used as a physical crosslinking agent, and the network structure and water absorption performance may be influenced by reversible borate-related interactions between borate ions and hydroxyl groups of GG (Figure 5c). When the amount of borax is increased from 0.1 g to 0.2 g, the water absorption capacity increases slightly. This increase may be associated with the formation of an appropriate dynamic crosslinking network, which may help stabilize the structure while retaining sufficient hydrophilic groups and suitable pore space for water uptake. However, when the amount of borax continues to increase, the water absorption decreases significantly. This decrease may result from an increase in dynamic crosslinking interactions, which may make the three-dimensional network more compact and reduce the accessibility of hydrophilic -OH groups, thereby limiting water entry and polymer-chain extension.

In the IAGG hydrogel system, temperature may affect the water absorption performance by regulating the hydration of hydrophilic groups, the thermal motion of the crosslinking network, and the stability of dynamic crosslinking interactions (Figure 5d). When the temperature rises from 50 °C to 70 °C, the water absorption capacity continues to increase. This increase may be related to enhanced permeability of water molecules and increased thermal motion of polymer chains, which may facilitate network expansion while maintaining effective hydration of hydrophilic groups. However, when the temperature rises from 70 °C to 80 °C, the water absorption of the hydrogel decreases significantly. This decrease may be associated with partial disruption of dynamic borate-related interactions, weakened hydrogen bonding between hydrophilic groups and water molecules, and excessive thermal motion that may induce relaxation or partial collapse of the crosslinked network structure. Thus, the overall trend first increases and then decreases.

**Figure 6:**
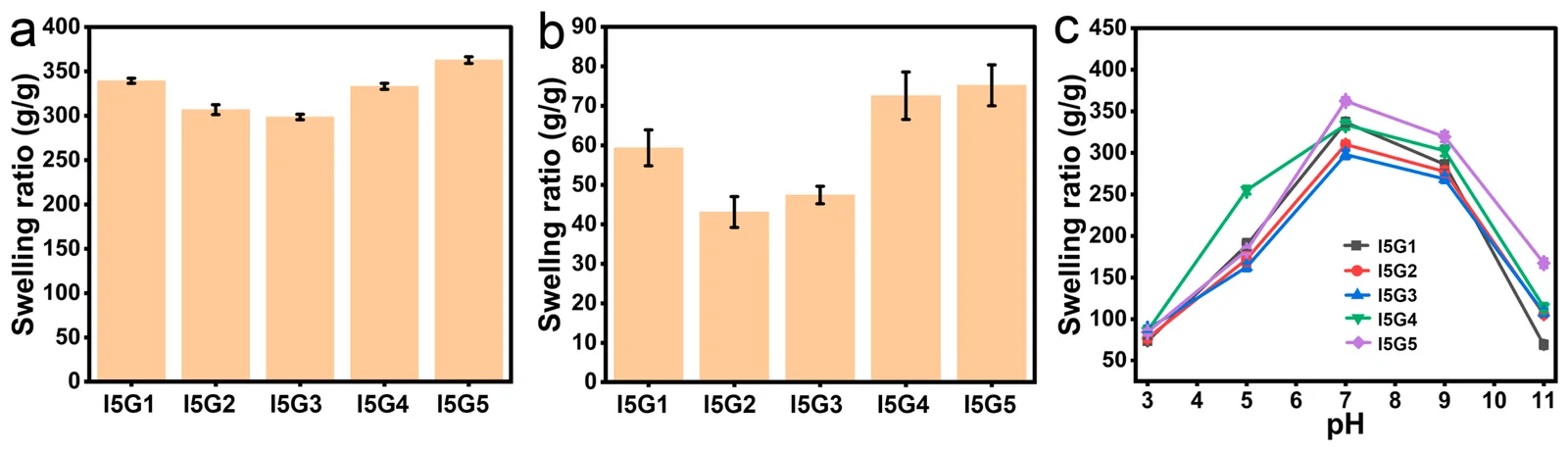
Water absorption of IAGG hydrogels in different environments: (**a**) in deionized water; (**b**) in 0.9% NaCl solution; (**c**) in water at different pH levels.

In deionized water, hydrogels with different IA/GG ratios all show high water absorption, which remains around 300 g/g as a whole (Figure 6a). With the increase in the GG ratio, the water absorption capacity decreases slightly at first and then increases; among these, the water absorption capacity of I5G3 is slightly lower, while that of I5G5 is the highest, indicating that increasing the GG ratio in a deionized water environment can introduce more hydrophilic groups such as hydroxyl groups, thus enhancing the water absorption capacity of the hydrogel. In 0.9% NaCl solution, the water absorption of all IAGG hydrogels is significantly lower than in deionized water, and it fluctuates obviously with the change of IA/GG ratio (Figure 6b). The water absorption of I5G1 is 58.5 g/g, that of I5G2 is reduced to 42.1 g/g, and that of I5G5 is increased to 74.5 g/g. This is because the ion shielding effect of salt solution weakens the electrostatic interactions of hydrophilic groups, and more hydroxyl groups introduced at a high GG ratio can retain part of the hydration ability in a salt environment, thus alleviating the adverse effects caused by ion shielding and improving water absorption. The water absorption of hydrogels increases first and then decreases with pH value (Figure 6c), and reaches a peak at pH = 7. When the pH is acidic or alkaline, the water absorption capacity decreases to different degrees, and the greater the deviation, the more significantly the water absorption capacity decreases. This phenomenon may be due to the fact that hydrophilic groups such as hydroxyl and carboxyl groups of the hydrogel have the strongest hydration under neutral conditions, while an acidic or alkaline environment will change the ionization state of carboxyl groups or destroy some crosslinked network structures [32], thus weakening the water absorption capacity of the hydrogel.

### 2.3. Water Retention Performance of IAGG Hydrogel

**Figure 7:**
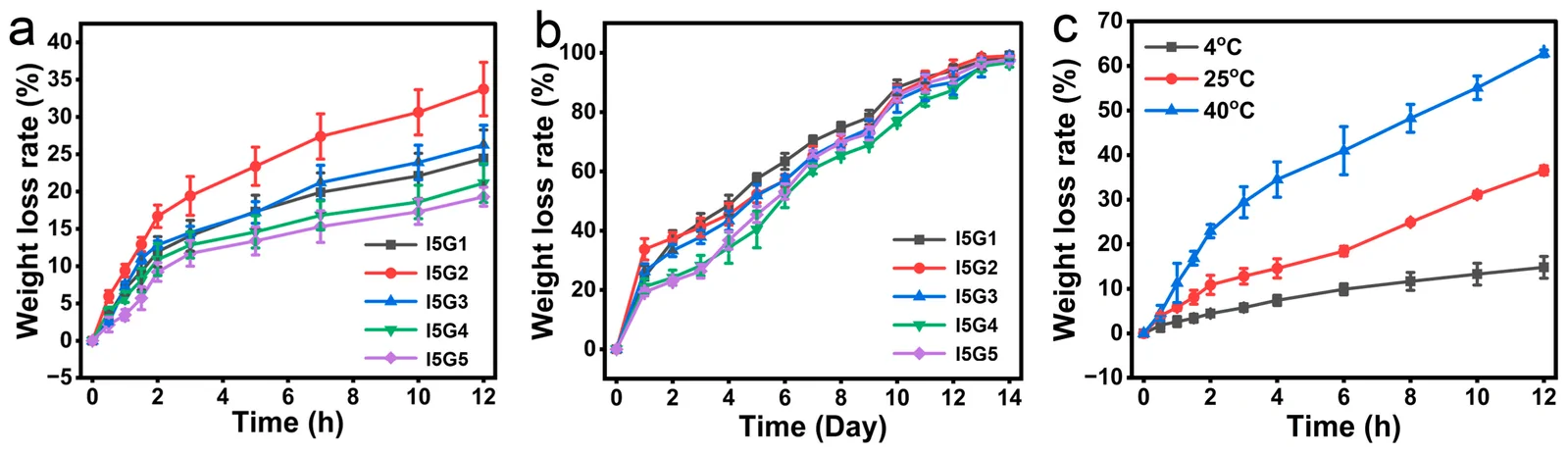
Water retention of IAGG hydrogel at 25 °C and 35% humidity: (**a**) within 12 h and (**b**) over 14 days; (**c**) Water retention of I5G2 hydrogel at different temperatures.

Water-retention performance is a key parameter for evaluating the practical applicability of hydrogels as soil water-retaining materials. In the short-term water retention test of 12 h, the mass loss rate of all IAGG hydrogels increased with time (Figure 7a). Among them, hydrogels with higher IA proportions exhibited faster mass loss, with a loss rate of 34% at 12 h, while the hydrogel with a lower IA ratio lost only 18%. Moreover, the long-term water-holding test for 14 days showed that the mass loss rate of all hydrogels increased continuously with time, and the loss rate was faster during the first 8 days and then gradually slowed down in the later period. By 14 days, the loss rate of all ratios was close to 100% (Figure 7b). Among them, the early loss rate of the hydrogel with a lower IA ratio is slower, indicating that its long-term water retention is slightly better than that of the hydrogel with a higher IA ratio.

In addition, the mass loss rates of I5G2 at 4 °C, 25 °C, and 40 °C all increased with time, showing obvious temperature dependence (Figure 7c). The 0–4 h period is the rapid dehydration stage, and after 4 h, it enters the slow dehydration stage; meanwhile, the mass loss rate follows the order 40 °C > 25 °C > 4 °C, which indicates that lower temperatures were more favorable for water retention by the hydrogel. A large number of water molecules are bound in the IAGG hydrogel network through hydrogen bonds and ionic bonds. An increase in temperature will aggravate the thermal motion of water molecules, weaken the interaction between the hydrogel network and water molecules, and accelerate the evaporation and diffusion of water, resulting in the decline of water retention performance [33].

### 2.4. The Reusable Performance of IAGG Hydrogel

**Figure 8:**
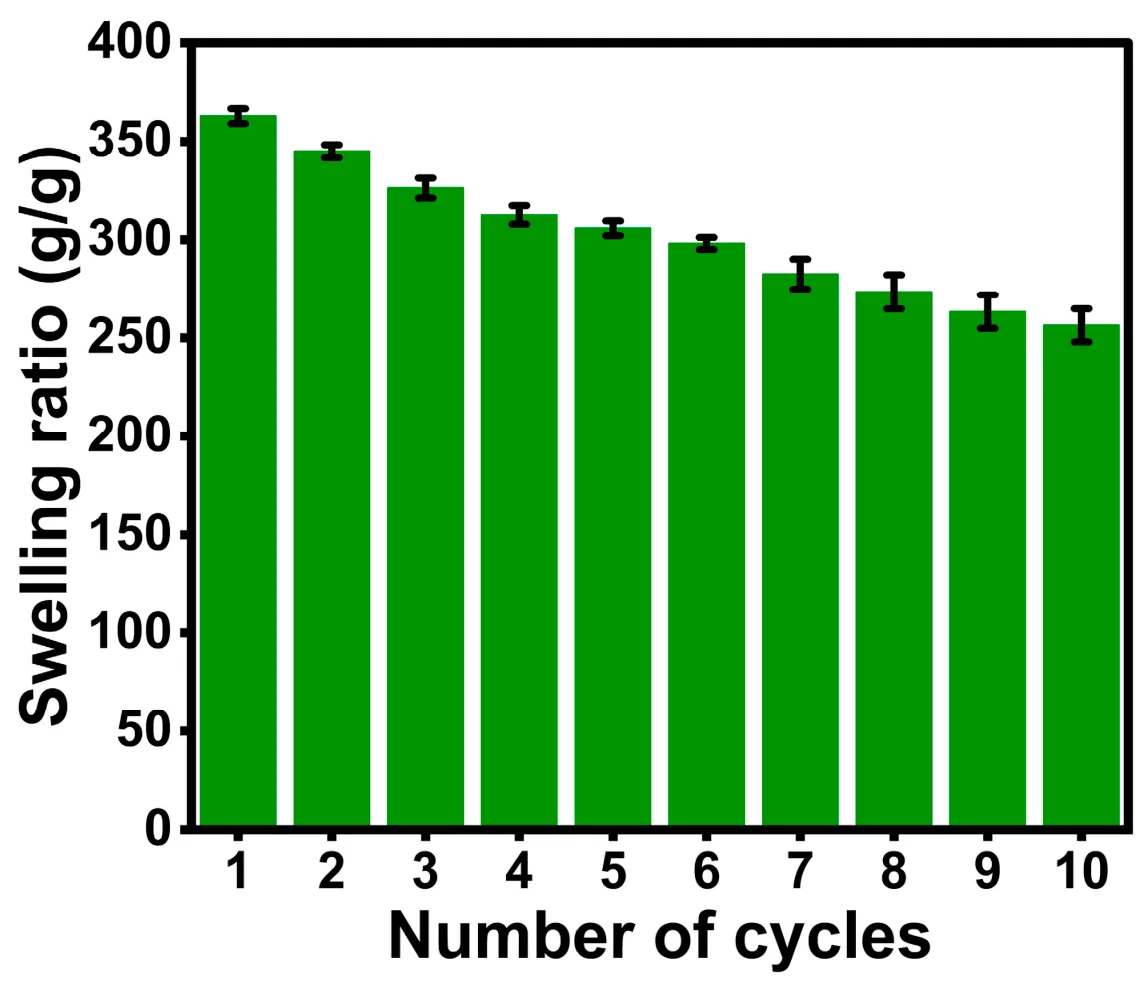
Water absorption capacity of I5G5 hydrogel during 10 repeated water absorption vacuum drying cycles in deionized water.

In order to evaluate the water absorption stability of IAGG hydrogel in the process of repeated use, 10 water absorption-dehydration-water absorption cycle tests were carried out. As can be seen from Figure 8, the water absorption capacity of the I5G5 hydrogel gradually decreased with increasing cycle number and remained at 255.4 g/g after the tenth cycle. These results indicate that the I5G5 hydrogel retained substantial water-absorption capacity after repeated water absorption-dehydration cycles and exhibited good reusability.

### 2.5. Application of IAGG Hydrogel in Soil

**Figure 9:**
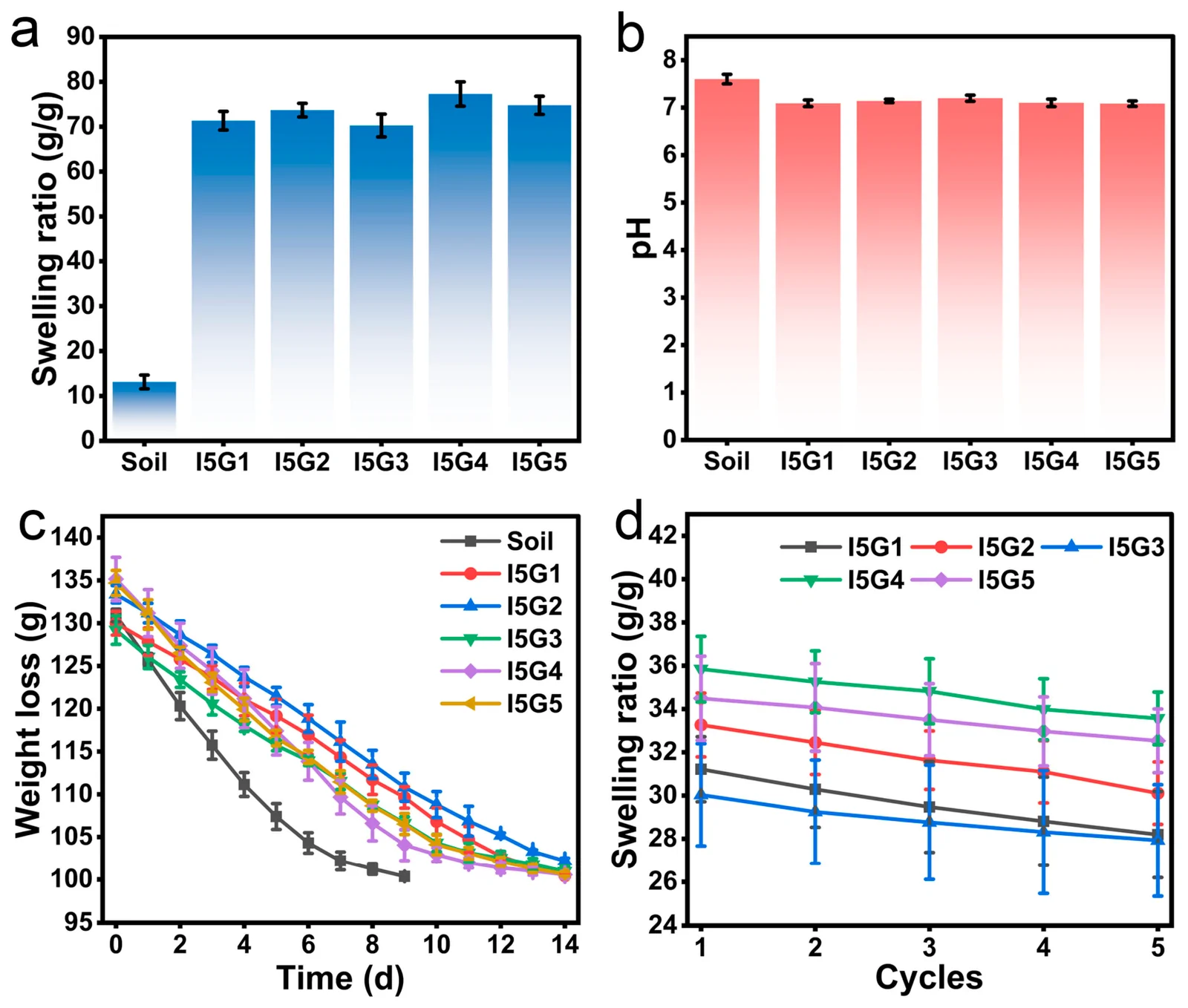
Mine soil and mine soil with added IAGG hydrogel: (**a**) Maximum water-holding capacity and (**b**) pH after adding deionized water; (**c**) Water retention over 14 days and (**d**) Water absorption capacity after five repeated watering cycles of mine soil with IAGG hydrogel at 25 °C.

Figure 9a shows the comparison of the maximum water-holding capacity between pure soil and soil with IAGG hydrogels of different proportions. The maximum water-holding capacity of pure soil is only 13.3 g/g, and the water-holding capacity of soil is significantly improved after IAGG hydrogel is added, and there are differences among different ratios. Among them, the maximum water-holding capacity of modified soil in group I5G4 reached its peak, which was 77.6 g/g, and that in group I5G5 was 74.3 g/g. Although the water-holding capacities of I5G1, I5G2, and I5G3 were lower than those of I5G4 and I5G5, they were still much higher than those of pure soil, which indicated that IAGG hydrogel could be used as an efficient soil water-holding agent, and the I5G4-treated soil exhibited the highest water-holding capacity. Moreover, the pH value of pure soil is about 7.6 (weakly alkaline); after adding hydrogel, the pH value of each group of modified soil decreased slightly and stabilized at 7.1–7.3, which was in the neutral to weakly alkaline range, and the difference in pH value among the groups was very small (Figure 9b). These results indicate that the addition of IAGG hydrogel only slightly altered the soil pH and did not change the overall acid-base environment of the soil, so it has good compatibility with the tested soil environment.

In addition, the weight loss rate of pure soil after 14 d at 25 °C is the fastest, and the water retention capacity is the worst. However, the weight loss rate of soil in each group slowed down significantly after adding IAGG hydrogel. Among them, group I5G5 had the least water loss, which extended the effective water retention time of soil from 5 d to 14 d (Figure 9c). This is because the three-dimensional network structure of IAGG hydrogel can effectively bind water, slow down the evaporation and loss of soil water, and the high proportion of the hydrogel can form a denser water-holding network, further improving the long-term water-holding capacity of soil. Besides, with the increase in the number of cycles from 1 to 5, the water absorption capacity of each group of modified soil decreased slowly but remained at a high level. The I5G4 group is the highest in each cycle, and it still maintains about 34 g/g after five cycles (Figure 9d), which indicates that the IAGG hydrogel-modified soil has good recycling stability.

**Figure 10:**
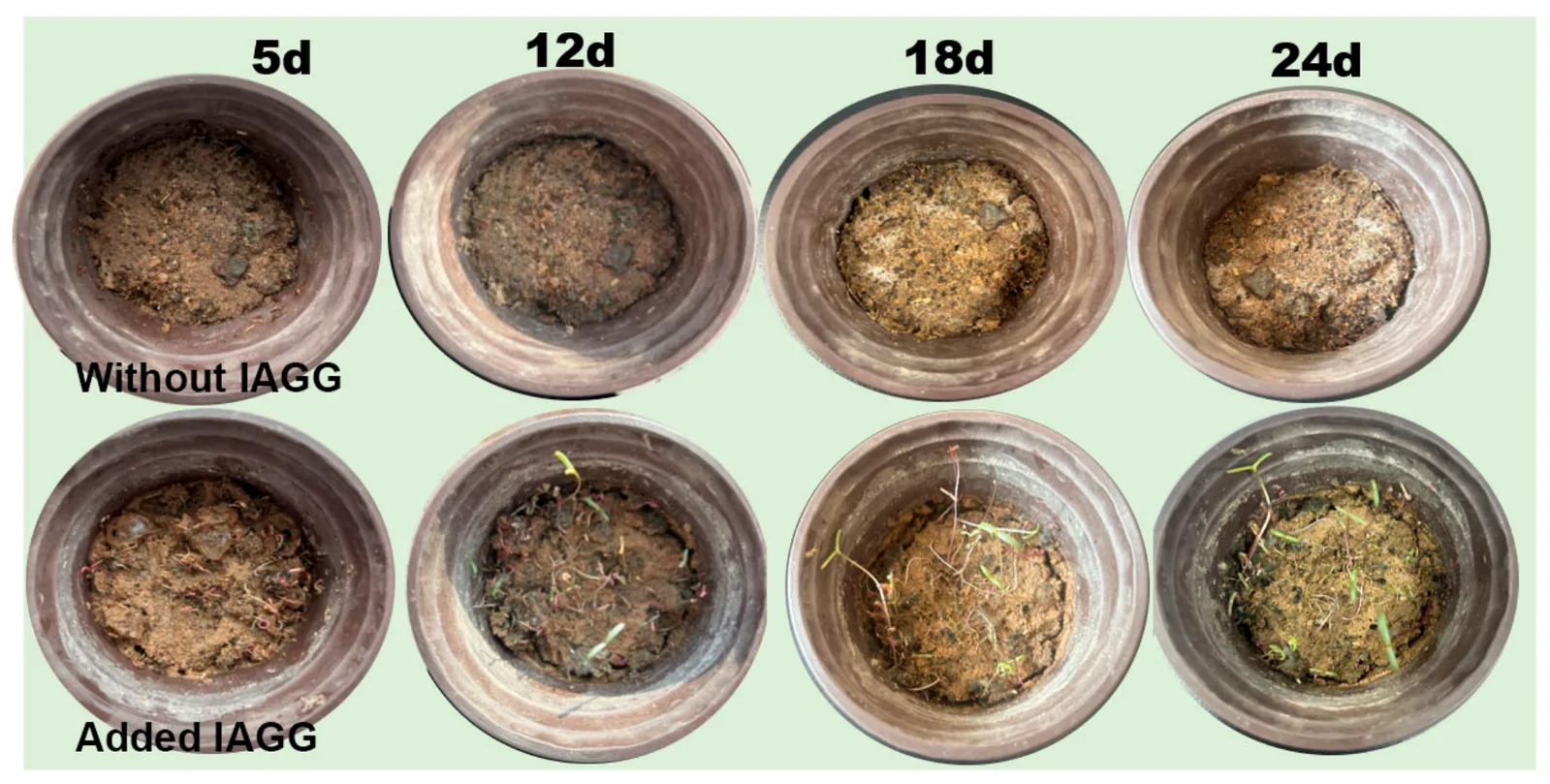
Growth observations of *Suaeda salsa* in mine soil at 5, 12, 18, and 24 d. The control group consisted of mine soil without IAGG hydrogel, whereas the IAGG-treated group contained I5G5 hydrogel mixed with the soil at a mass ratio of 1:50 (hydrogel:soil).

Figure 10 shows differences in the growth dynamics of *Suaeda salsa* in mine soil at 5, 12, 18, and 24 d without and with IAGG hydrogel. In the mine soil without hydrogel, no seed germination was observed throughout the observation period, and the soil gradually became dry and compact. This behavior may be related to the limited water-retention capacity of the untreated mine soil, which was unable to maintain a favorable moisture environment for seed germination. In contrast, seedling emergence was observed in the IAGG-treated mine soil on day 12, followed by continued seedling development on days 18 and 24, including further development of stems and leaves. These observations suggest that incorporation of IAGG hydrogel improved the water-retention environment of the mine soil and provided more favorable conditions for seed germination and subsequent seedling development.

### 2.6. Soil-Burial Degradation Behavior of IAGG Hydrogel

**Figure 11:**
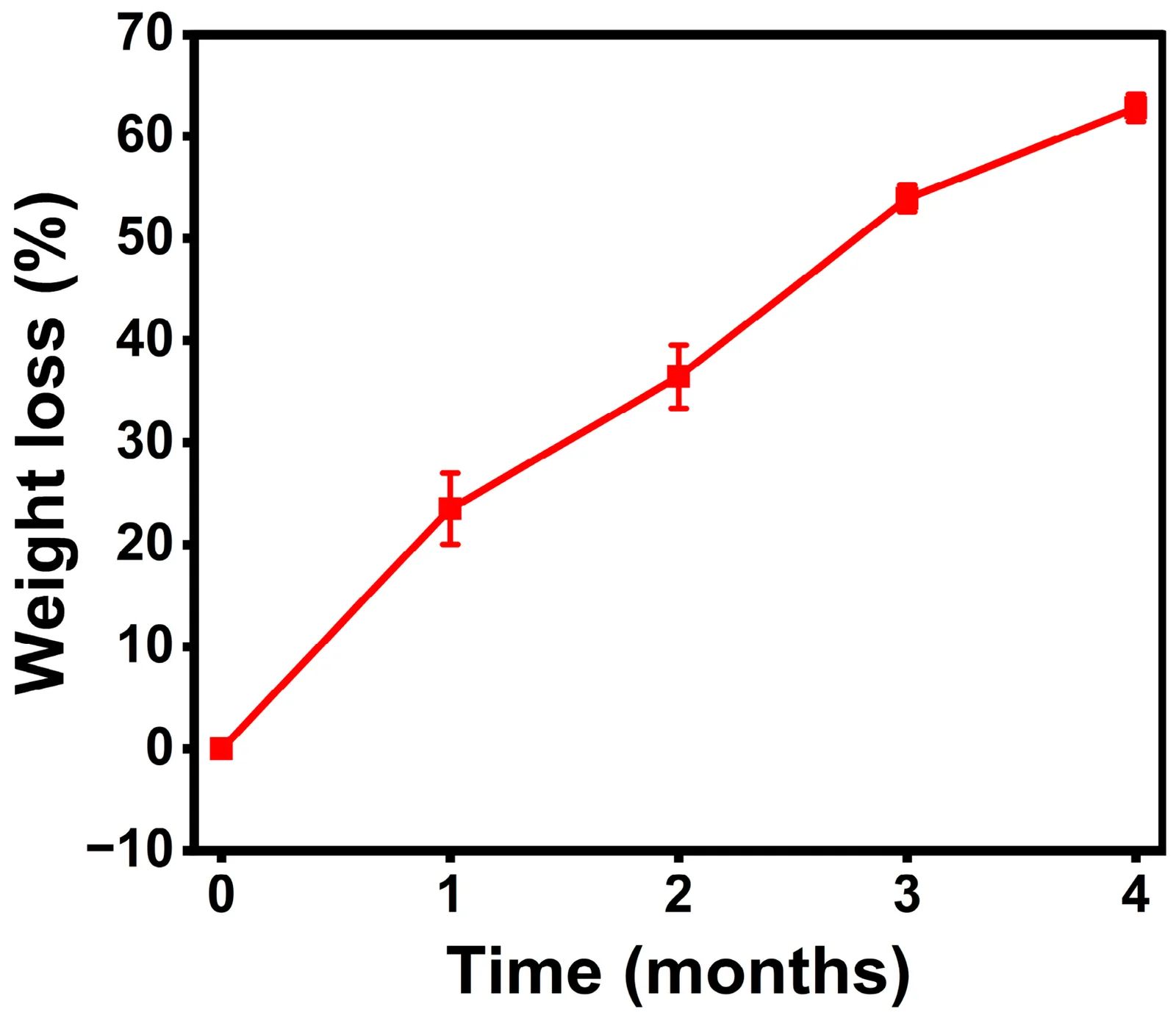
Weight loss of IAGG hydrogel during soil burial at 0, 1, 2, 3, and 4 months.

With the extension of the degradation time from 0 months to 4 months, the weight loss of the IAGG hydrogel increased continuously, reaching approximately 63% after 4 months (Figure 11), indicating substantial mass loss under the tested soil-burial conditions. During the initial stage, the weight loss increased relatively slowly, which may be associated with the release of soluble low-molecular-weight components and the gradual disruption of relatively weak interactions within the hydrogel network. From 1 to 4 months, the increase in weight loss became more pronounced, suggesting progressive deterioration of the crosslinked network and loss of polymeric components. Because the soil-burial test reflects overall mass loss, these changes may involve a combination of biological and physicochemical processes.

**Figure 12:**
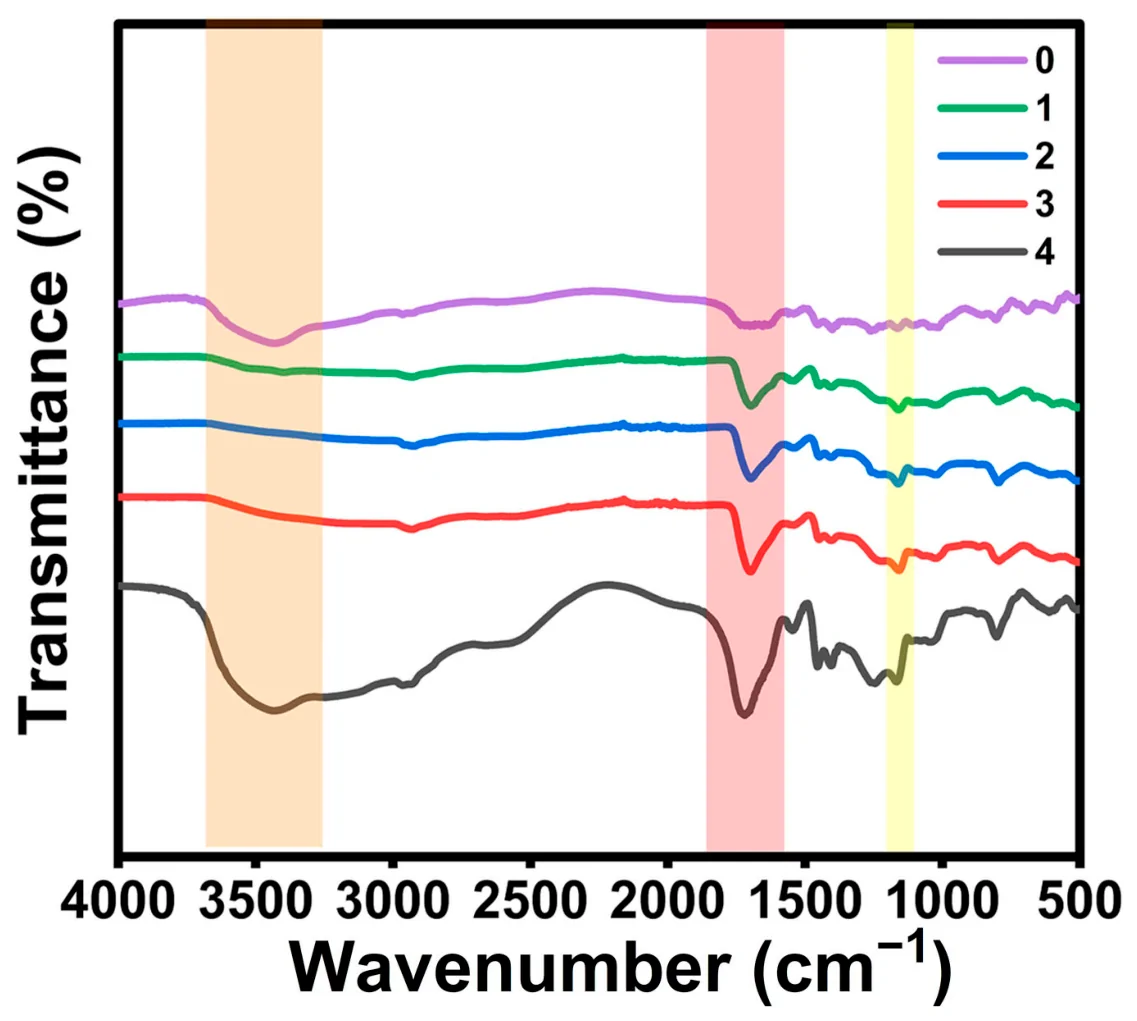
FTIR spectra of IAGG hydrogel after soil burial for 0, 1, 2, 3, and 4 months. The labels 0, 1, 2, 3, and 4 correspond to the respective soil-burial times in months.

Compared with the FTIR spectrum before soil burial, the intensities of several characteristic bands changed progressively with increasing burial time (Figure 12). The intensity of the -OH peak in the region of 3000–3500 cm^−1^ is weakened, which may be associated with changes in hydroxyl-containing structures during soil burial. The intensity of the C=O-related peak in the region of 1500–1700 cm^−1^ decreased, suggesting possible disruption of ester-related structures. The C-O-C ether bond peak in the region of 1000–1200 cm^−1^ decreased [34], which may reflect progressive changes in the polysaccharide-based network structure during soil burial.

## 3. Conclusions

This study used GG as the base material and IA as the functional monomer. By combining IA-related covalent interactions, ECH-mediated ether crosslinking, and borax-mediated dynamic interactions, an IAGG composite hydrogel with a covalent–dynamic multiple crosslinking network was prepared. FTIR analysis supported the formation of ester- and ether-related structures within the hydrogel network, while borax was considered to contribute to dynamic crosslinking interactions. SEM images showed a three-dimensional porous structure that became progressively more open as the GG proportion increased. Under optimal preparation conditions (IA/GG = 5:5, pH = 8, ECH 4 mL, borax 0.2 g, 70 °C, 4 h), the hydrogel had a water absorption of 370.7 g/g in distilled water and 74.5 g/g in 0.9% NaCl solution. After 10 cycles, the I5G5 sample retained a water absorption capacity of 255.4 g/g. Soil application tests showed that IAGG increased the maximum water retention of mine soil from 13.3 g/g to 77.6 g/g and extended the effective water retention time from 5 days to 14 days. In the preliminary planting experiment, seedling emergence and subsequent development of *Suaeda salsa* were observed in the I5G5-treated soil, whereas no germination was observed in the untreated control during the observation period. After 4 months of soil burial, the hydrogel exhibited approximately 63% weight loss, indicating substantial structural deterioration under soil-burial conditions. These results suggest that the IAGG hydrogel has potential as a water-retention material for mine ecological restoration and provide a reference for the chemical modification and structural design of natural polysaccharide-based hydrogels.

## 4. Materials and Methods

### 4.1. Materials

Guar gum (GG, 99% purity) was purchased from Beijing Guaran Technology Co., Ltd. (Beijing, China); its average molecular weight was approximately 2.2 × 10^6^ g/mol, and the viscosity of a 1 wt% aqueous solution at 25 °C was 5000–5500 cP at pH 6–8. Sodium tetraborate decahydrate (Na_2_B_4_O_7_·10H_2_O, 99.5% purity) and sodium hydroxide (NaOH, 98% purity) were purchased from Tianjin Xinbote Chemical Co., Ltd. (Tianjin, China); itaconic acid (IA, 99% purity) was obtained from Shanghai Macklin Biochemical Technology Co., Ltd. (Shanghai, China); epichlorohydrin (ECH, 99% purity) was obtained from Tianjin Xinbote Chemical Co., Ltd. (Tianjin, China); HCl solution (36 wt%) was purchased from Hongyan Reagent Factory (Tianjin, China), Hedong District, Tianjin; sodium chloride (NaCl, 99.5% purity) was purchased from Tianjin Zhiyuan Chemical Reagent Co., Ltd. (Tianjin, China); and nitrogen (N_2_, 99.99% purity) was purchased from Tianjin Yongsheng Fine Chemical Co., Ltd. (Tianjin, China).

### 4.2. Preparation of IAGG Hydrogel

**Figure 13:**
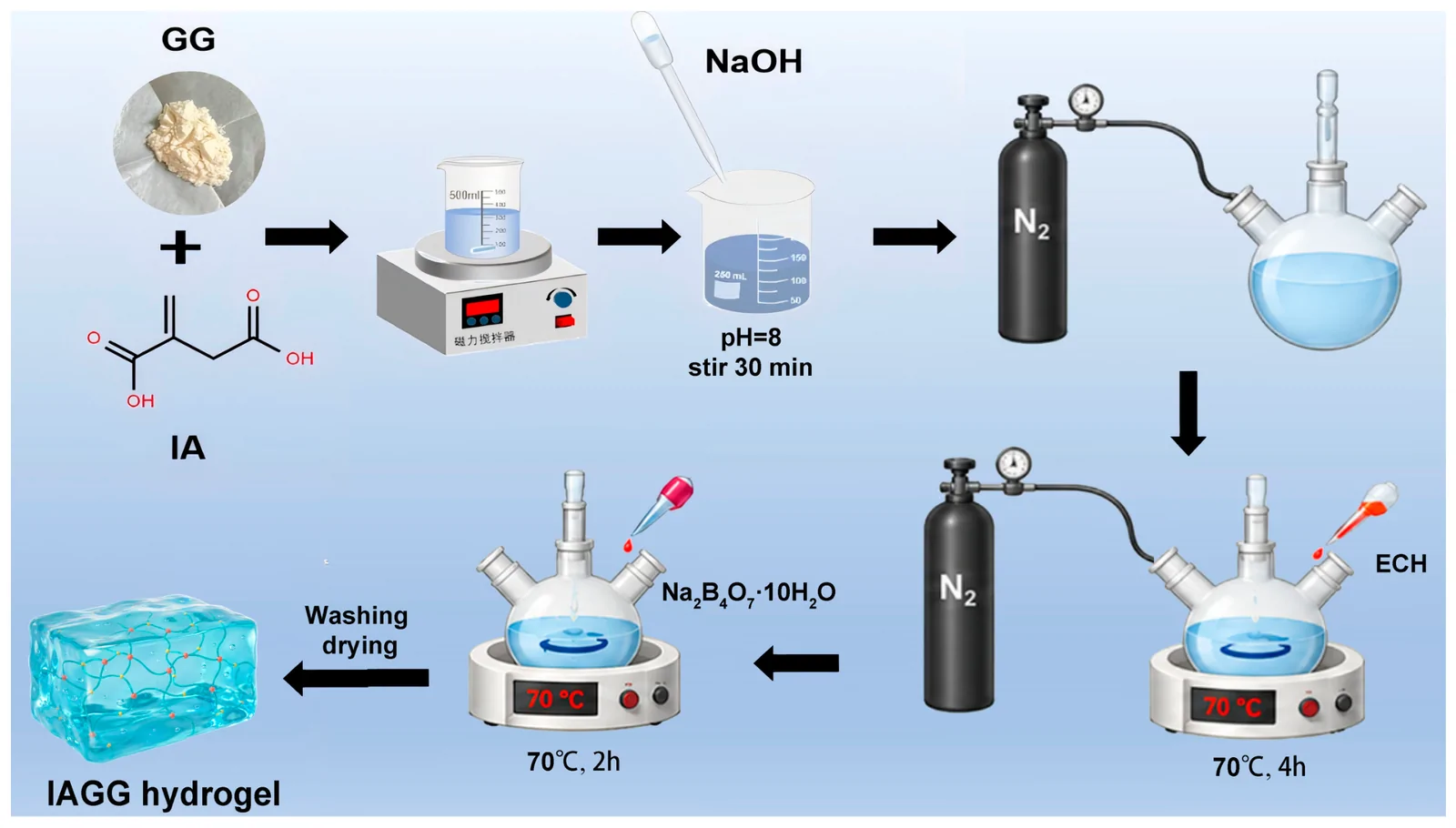
Schematic diagram of preparation of IAGG hydrogel.

The preparation procedure of the IAGG hydrogel is schematically illustrated in Figure 13. For the I5G5 formulation, 0.5 g of GG was weighed into a beaker and dissolved in 50 mL of deionized water under stirring, while 0.5 g of IA was dissolved in 20 mL of deionized water in a separate beaker. The GG solution and IA solution were transferred into a three-neck flask and continuously stirred magnetically at a constant temperature until uniform, forming a homogeneous and transparent mixed solution. Subsequently, the NaOH solution was added dropwise to the above solution to adjust the pH to 8, and the mixture was further stirred for 30 min to ensure uniform mixing. The three-neck flask was then connected to a nitrogen protection device, and high-purity nitrogen was continuously introduced into the system to exhaust the air and maintain a nitrogen atmosphere. The temperature was raised to 70 °C, and 4 mL of epichlorohydrin was accurately measured and slowly added dropwise into the mixed solution, followed by reaction for 4 h under constant-temperature stirring. Then, a borax solution (0.2 g of borax dissolved in 10 mL of deionized water) was added to the system, and the reaction was continued for 2 h under the same conditions to obtain the crude IAGG hydrogel product. The product was repeatedly washed with deionized water until the washing liquid became neutral. The hydrogel was dried in a vacuum drying oven at 80 °C to obtain the dried hydrogel. After drying, grinding, and sieving, the final itaconic acid-modified guar gum composite hydrogel (IAGG hydrogel) was obtained. For the formulation-comparison series, the IA amount was fixed at 0.5 g, and the GG amount was adjusted to 0.1, 0.2, 0.3, 0.4, and 0.5 g to obtain IA/GG mass ratios of 5:1, 5:2, 5:3, 5:4, and 5:5, respectively. These samples were denoted I5G1, I5G2, I5G3, I5G4, and I5G5, where “I” and “G” represent IA and GG, and the numbers indicate their relative mass ratio. The detailed compositions are summarized in Table 1.

### 4.3. Characterization

Fourier transform infrared spectroscopy (FTIR, Thermo Fisher Scientific Nicolet iS20, Madison, WI, USA) was used to analyze the functional groups of the materials. KBr pellets were prepared, and the infrared characteristic absorption peaks were recorded in the range of 400–4000 cm^−1^. Thermogravimetric analysis (TGA, TA SDT Q600, New Castle, DE, USA) was performed under a nitrogen atmosphere at a heating rate of 10 °C/min from 40 °C to 800 °C. Derivative thermogravimetric (DTG) curves were obtained from the corresponding TGA data. Scanning electron microscopy (SEM, Zeiss Sigma 300, Oberkochen, Germany) was used to observe the micro-morphology of the materials. Rheological analysis of the hydrogels was carried out using a rotary rheometer (TA DHR-2, New Castle, DE, USA) with a parallel plate diameter of 40 mm, a gap height of 1000 μm, an oscillation frequency of 0.01–100 Hz, an oscillation stress of 0.05 Pa, and a test temperature of 25 °C.

### 4.4. Water Absorption Test of IAGG Hydrogel

In water: A certain amount of dry hydrogel was placed into solutions with different pH values (3, 5, 7, 9, and 11) and a 0.9% NaCl solution for water absorption experiments. The mass change of the hydrogel was recorded at each time interval, and the weight was continuously measured until a constant weight was reached. The water absorption capacity of the hydrogel was calculated using the swelling ratio given in Equation (1) [35]:

(1) $$\mathrm{Swelling}\;\mathrm{ratio}=\frac{W_{s}-W_{1}}{W_{1}}$$

*W*_s_ (g) is the weight of the expanded hydrogel, and *W*_1_ (g) is the initial weight of the dried hydrogel.

Mine soil: Topsoil was collected from the mining area, and impurities such as stones and weeds were removed. The soil was completely dried, ground, and sieved through a 2 mm screen before use. The dried mine soil and dried hydrogel were weighed and uniformly mixed to obtain a hydrogel–soil mixed sample. The mixed samples were placed into a plastic cup lined with filter paper, and deionized water was slowly added into the cup while stirring until the samples were completely saturated. The samples were then left to stand and soak for 24 h to ensure that both the soil and hydrogel fully absorbed water. A pure soil system without hydrogel was used as a blank control. Three parallel experiments were conducted for each gradient group, and the average value was taken. The calculation of maximum water-holding capacity is shown in Equation (2):

(2) $$\mathrm{Maximum}\;\mathrm{water}\;\mathrm{holding}\;\mathrm{capacity}=\frac{W_{S1}-W_{1}-W_{2}-W_{3}}{W_{2}}$$

where W_s1_ (g) is the weight of the total mass in the saturated state; W_2_ (g) is the weight of mineral soil, and W_3_ (g) is the weight of the plastic cup and filter paper.

### 4.5. Test of Water Retention Performance of IAGG Hydrogel

In the air: IAGG hydrogel was placed in a beaker filled with deionized water, and experiments were carried out at low temperature (4 °C), room temperature (25 °C), and high temperature (40 °C). The data were continuously recorded until 12 h. Meanwhile, the water retention capacity of IAGG hydrogel was measured at 25 °C and 35% relative humidity for 14 days. Each condition was tested in triplicate. The calculation method of weight loss is shown in Equation (3):

(3) $$\mathrm{Water}\;\mathrm{retention}\;\mathrm{capacity}\;(\mathrm{air})=\frac{W_{t}-W_{1}}{W_{S}-W_{1}}$$

where W_t_ (g) represents the weight of IAGG hydrogel on day t.

In mine soil: The obtained IAGG powder was thoroughly mixed with dry soil at a mass ratio (*w*/*w*) of 1:50 (IAGG:soil). The soil and IAGG powder, with an initial weight of about 50 g, were placed into a plastic cup (top diameter 8 cm, height 9.2 cm). The sample was saturated with 100 mL of water, and the temperature was controlled at 25 °C with a relative humidity of 35%. The weight of each sample was recorded at the relevant time points. Each condition was repeated three times to determine the water retention rate of the samples at each time point. The calculation method of water retention capacity is shown in Equation (4):

(4) $$\mathrm{Water}\;\mathrm{retention}\;\mathrm{capacity}\;(\mathrm{mine}\;\mathrm{soil})=\frac{W_{t}-W_{1}-W_{2}-W_{3}}{W_{2}}$$

where W_t_ (g) is the total sample weight at a certain time.

### 4.6. IAGG Hydrogel Recycling Test

To evaluate the recycling performance of the IAGG hydrogel, the I5G5 hydrogel was selected for the recycling test. The dried I5G5 hydrogel was first subjected to a water absorption test in deionized water. After water absorption, the swollen hydrogel was vacuum-dried at 60 °C for 10 h and then subjected to the next water absorption test. This water absorption–vacuum drying process was repeated for a total of 10 cycles. The water absorption capacity was determined after each cycle. Each condition was tested in triplicate, and the average value was reported.

### 4.7. Planting Experiment

To preliminarily evaluate the potential effect of IAGG hydrogel powder on seed germination and seedling development under mine soil conditions, a qualitative planting experiment was conducted indoors at room temperature. Soil samples were collected from the Shitoumei No. 1 open-pit coal mine in the Santanghu mining area, Barkol Kazakh Autonomous County, Hami City, Xinjiang, China. The I5G5 hydrogel was uniformly mixed with the soil at a mass ratio of 1:50 (hydrogel:soil). Five *Suaeda salsa* seeds were sown in each pot at a depth of 0–3 cm. At the beginning of the experiment, water equivalent to 30 wt% of the soil mass was added once, and no additional water was supplied during the subsequent observation period. Photographs were taken at predetermined time points to record seed germination and subsequent seedling development. This experiment was intended as a preliminary qualitative observation and was not designed with independent replicate pots for statistical analysis. Accordingly, the planting results are interpreted only as qualitative observations, and further replicated experiments with quantitative plant-growth measurements are required for comprehensive evaluation.

### 4.8. Soil-Burial Degradation Test

The degradation behavior of the IAGG hydrogel under soil-burial conditions was evaluated using a soil-burial test. The soil used in this experiment was collected from the Shitoumei No. 1 open-pit coal mine in the Santanghu mining area, Barkol Kazakh Autonomous County, Hami City, Xinjiang, China. A fixed amount of hydrogel was placed in a nylon net bag (60 mesh) and buried 6 cm deep in the soil. Samples were recovered after 1, 2, 3, and 4 months of burial, with the initial sample at 0 months used as the reference. Each condition was tested in triplicate. After recovery, the samples were gently rinsed with water to remove adhering soil particles and then dried at 60 °C for 4 h before weighing. The mass loss of the hydrogel after soil burial was calculated from the difference between the initial dry mass and the recovered dry mass. The weight loss (%) was calculated according to Equation (5):

(5) $$\;\mathrm{Weight}\;\mathrm{loss}\;(\%)=\frac{M_{0}-M_{t}}{M_{0}}\times 100\%$$

where *M*_0_ (g) is the initial dry weight of the hydrogel sample, *M*_t_ (g) is the dry weight of the recovered sample after degradation, and t is the degradation time.

## Figures and Tables

**Table 1:** Composition and designation of IAGG hydrogels with different IA/GG mass ratios.

Sample	IA/GG Mass Ratio	IA Amount (g)	GG Amount (g)	ECH (mL)	Na_2_B_4_O_7_·10H_2_O (g)
I5G1	5:1	0.5	0.1	4	0.2
I5G2	5:2	0.5	0.2	4	0.2
I5G3	5:3	0.5	0.3	4	0.2
I5G4	5:4	0.5	0.4	4	0.2
I5G5	5:5	0.5	0.5	4	0.2

## Data Availability

The data presented in this study are available from the corresponding author upon reasonable request.
